# Beef Color Standard, including marbled Wagyu beef, determined using a portable near-infrared optical fiber spectrometer

**DOI:** 10.5713/ab.251019

**Published:** 2026-06-01

**Authors:** Kazunori Matsumoto, Naoaki Obana, Masakazu Irie

**Affiliations:** 1 National Livestock Breeding Center, Nishigo, Japan

**Keywords:** Beef Carcass, Meat Color, Near-infrared Optical Fiber Method, Portable Optical Device

## Abstract

**Objective:**

The purpose of this study was to see if a portable near-infrared optical fiber spectrometer with a wavelength range of 700–1,050 nm could objectively predict the Beef Color Standard (BCS), which is visually evaluated during beef carcass grading.

**Methods:**

A total of 1,077 carcasses were procured from Japanese Black (JB) Wagyu (n = 534), crossbred (F1) (n = 367), and dairy cows (n = 176) shipped to meat markets. The cut surface between the sixth and seventh ribs was visually evaluated by grading members using the BCS. In addition, a near-infrared optical fiber spectrometer was applied to the cut surface to obtain the reflectance spectrum. Using data from 827 carcasses, a calibration model was established through a partial least squares regression analysis using the BCS value as the objective variable, while the optical data following quadratic differential processing as the explanatory variable. Using data from 250 additional animals, the calibration model and predicted BCS values were verified for accuracy.

**Results:**

The calibration model exhibited a high correlation coefficient of 0.83 and a standard error of calibration of 0.70. The predicted BCS by near-infrared evaluation rarely differed significantly from the actual BCS, and the difference was roughly distributed in the range of ±1. For verifying the accuracy of our calibration model, the correlation coefficient between the actual BCS and the predicted value for the validation sample was 0.75, and the standard error of prediction was 0.71. The probability that the error between the measured and optically predicted BCS values in the validation samples was within ±1 and ±0 was 97.6% and 54.4%, respectively.

**Conclusion:**

Using a portable near-infrared fiber optic spectrometer, we demonstrated that the BCS value of beef carcasses, particularly marbled Wagyu beef in meat markets, can be objectively evaluated with relatively high accuracy.

## INTRODUCTION

In a survey of beef purchasing sentiment in Japan, consumers considered the appearance of the meat, such as color and shine, more valuable after price and expiration date and ranked it above the degree of marbling and brand name [1]. Meat color of beef is graded from 1 to 7 according to the Beef Color Standard (BCS) for meat quality grading by the Japan Meat Grading Association (JMGA). The BCS standard is based on the silicon meat color standard model created by Nakai [2] at the Livestock Experiment Station of the Ministry of Agriculture, Forestry, and Fisheries in 1985.

When measuring meat color in the laboratory, L*, a*, and b* values are determined using a colorimeter. For example, in studies on meat color, PSE pork, DFD pork [3], and meat color changes associated with metmyoglobin formation in beef during shelf life [4,5] have typically used L*, a*, and b* values; however, if the meat color with high marbling is evaluated with a colorimeter, it is not an accurate indicator of meat color because the fat color interferes with the measurement. Thus, the meat color judged by the JMGA standard and the numerical value obtained from the colorimeter do not match well. To address this limitation, near-infrared (NIR) spectroscopy measurement may be able to objectively evaluate meat color, including marbling [6]. Objective predictions of BMS (Beef Marbling Standard) numbers in Japan and marbling scores overseas, which are integer values like BCS, have been reported by image analysis [7–10]. Since rating by humans requires long working hours in refrigerated rooms, it is necessary to develop objective methods using evaluation equipment. In addition, since the rating results have a strong impact on carcass prices, it is necessary to develop technologies that help minimize the discrepancy between the equipment-predicted values and those actually measured by the rating officers [8,11]. For the above reasons, we consider the NIR method suitable for an objective evaluation of BCS.

The nondestructive measurement technology for meat using a portable NIR optical fiber spectrometer is widely used in Japan to assess the fat quality of beef [12] and pork carcasses [13]. More than 200 units of this device (Fat Analyzer, S-7040; Soma Optical) have already been sold in Japan, and the JMGA evaluates the fat quality of more than 10,000 samples of beef and pork carcasses annually. This device enables the fast, safe, and inexpensive nondestructive measurement of meat quality in carcass lines by emitting light with a wavelength of 700–1,050 nm from an optical fiber. The light that penetrates, scatters, and reflects inside the meat is detected by another optical fiber and converted into data at every 1 nm. The data provides various physicochemical information about meat, which includes not only fat quality, but also the general components (moisture, fat, and protein) of pork and beef can be predicted [14]. The calibration software for each model can be installed inside the device and switched at start-up. Other meat quality calibration models suitable for NIR analysis can also be installed.

This device includes the NIR region and a wavelength range of 700–780 nm, which includes the red visible light required for meat color assessment. Therefore, optical data obtained from red meat and the amount of fat that affects meat color may be used for beef evaluation, including marbling. The evaluation of meat color is highly objective for experts, such as rating members of the JMGA; however, it may fluctuate for ordinary evaluators. Therefore, to establish an objective meat color evaluation method focusing on marbled Wagyu beef, a portable NIR optical fiber spectrometer was used to evaluate the meat color of beef carcasses.

## MATERIALS AND METHODS

### Sample collection

In this study, 1,077 carcasses of Japanese Black breed (JB, n = 534), crossbreed (F1, n = 367), and dairy cows (n = 176) were procured from seven meat markets and meat centers in Japan. All carcasses were subjected to BCS determination and optical measurement using a NIR optical fiber spectrometer.

### Beef Color Standard determination

**Figure 1:**
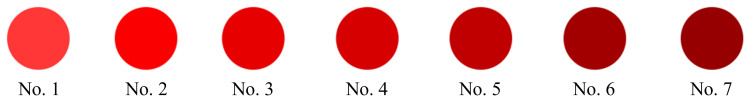
Beef Color Standard used in ratings by Japan Meat Grading Association.

The beef was evaluated by the BCS (Figure 1) by several rating members of the JMGA for the surface based on the 6th and 7th thoracic vertebrae of the carcasses.

### Beef Color Standard predicted by near-infrared measurement

**Figure 2:**
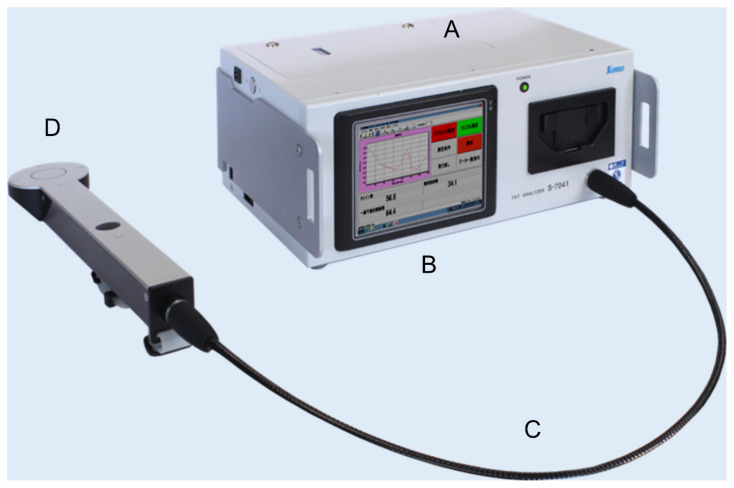
Handheld fiber optic near-infrared spectrometer (Fat Analyzer S-7040 type lipid measuring device; Soma Optical). (A) Main body, (B) display, (C) optical fiber, (D) probe.

A handheld fiber optic NIR spectrometer (Fat Analyzer, S-7040 type lipid measuring device; Soma Optical) was used to measure the fat quality of the beef (Figure 2). The spectrometer utilizes a specialized interactive probe, which is applied to the surface of the intact meat. It is based on a linear array method with a wavelength of 700–1,050 nm, and each measurement includes automatic scanning of 10 times per spectrum, which is completed within a few seconds. NIR measurements were made thrice at each location, and the average values were calculated for the spectral data.

### Statistical analysis

Unscrambler X (ver. 10.3; Kamo Software Japan) was used to process and analyze the NIR data. The absorbance spectra from 700 nm to 1,050 nm in NIR measurements were subjected to the Savitsky Gorei method [15]. Using the secondary differential spectra as the explanatory variable (X) and the BCS evaluation value by the grader as the objective variable (Y), the calibration model was generated by partial least squares regression (PLSR) analysis. Conventionally, the PLSR method is used for the preparation of calibration curves by NIR, and it is evolved from the principal component analysis method. The inherent hypothetical factors in the optical data were used as explanatory variables, and the factors were extracted from those that were highly correlated with the target variables. The factors were extracted repeatedly until a calibration curve with sufficient performance was obtained [16]. The number of factors in the calibration model was calculated using the optimal number of factors calculated using Unscrambler X.

A calibration model was established using 827 selected samples from all samples (n = 1,077). The remaining 250 samples were used as unknown samples to verify the accuracy of the calibration model. Thus, the optical measurements were fitted into the calibration model, and after obtaining the predicted BCS values, the accuracy of the measured and predicted BCS values was verified.

### Accuracy evaluation index based on the difference between the measured and predicted Beef Color Standard

Since BCS is an integer, in addition to verifying NIR accuracy with unknown samples, we also evaluated the same based on the variation calculated via “Measured BCS − Predicted BCS.” A proportion of individuals with a difference of 0, ±1, and ±2 within the sample for accuracy verification (n = 250) was used as an accuracy evaluation index following the method of Miyata and Kuchida [9].

## RESULTS AND DISCUSSION

### Distribution of Beef Color Standard number

**Figure 3:**
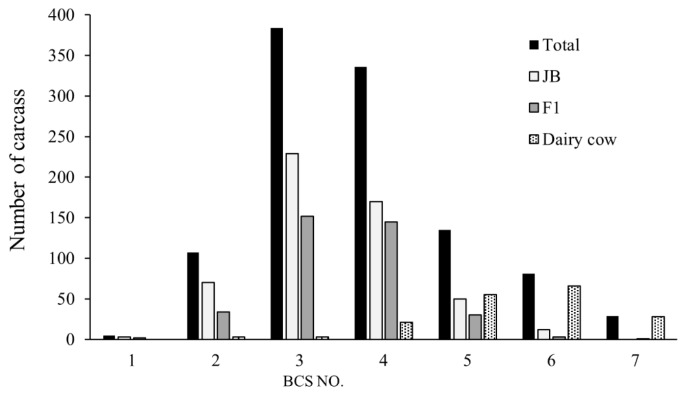
Distribution of the carcasses based on Beef Color Standard (BCS) No. from JB, F1, dairy cow, and the total. JB, Japanese Black; F1, cross breed.

Figure 3 shows a histogram of the BCS numbers obtained by visual evaluation of the rating members for all samples (n = 1,077). Although it was generally on the left, a wide range of sampling was achieved. The population of BCS No. 1 was very small, whereas BCS No. 7 was not evident in the JB carcass sampling. The average BCS numbers for JB and F1 were 3.4 and 3.5, respectively, which were similar to that of the 2024 national data (JB: 3.8, F1: 4.0, both average values) [17]. BCS Nos. 6 and 7 were more common in dairy cows. To establish a calibration model by NIR spectroscopy, the analysis items must be distributed over a wide area [18]. The BCS dataset in the present study was sufficiently broad to create a calibration model by NIR.

### Beef Color Standard calibration model

**Figure 4:**
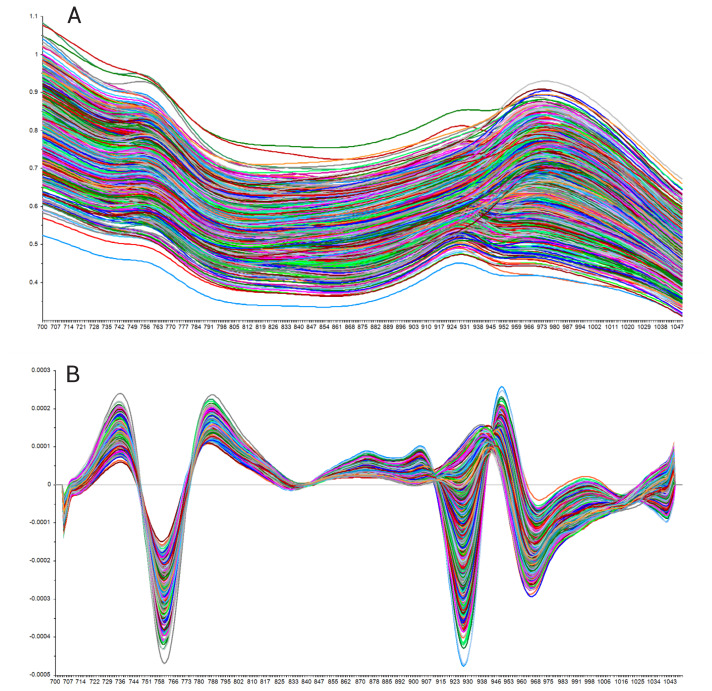
Spectra of the surface based on the 6th and 7th thoracic vertebrae from JB Wagyu carcasses by portable fiberoptic near-infrared spectrometer, showing raw reflectance spectra (A) and the 2nd derivative spectra (B). JB, Japanese Black.

NIR absorption spectra for the measurement location of the carcasses are shown in Figure 4. Protospectra (Figure 4A), as previously reported [14], showed a gradual curve. Additionally, the effects of baseline changes were reduced by secondary differentiation, and the effects of several sharp peaks were observed at the second derivative spectra (Figure 4B). Abnormal waveforms were not observed in any samples.

**Figure 5:**
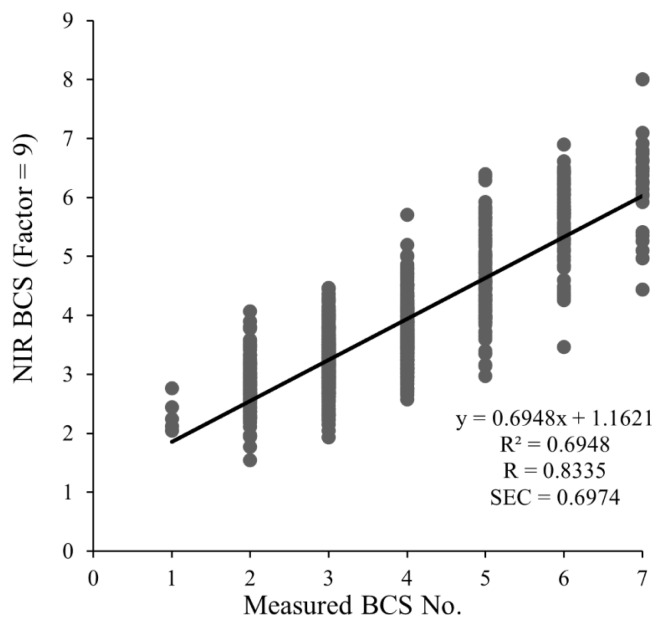
The calibration model for Beef Color Standard (BCS) in beef carcasses (n = 827), integrated into the handheld fiber optic near-infrared (NIR) spectrometer. R^2^, coefficient of determination; R, correlation coefficient; SEC, standard error of calibration.

The calibration model of BCS for meat color through NIR evaluation is shown in Figure 5. The model exhibited a high correlation (R) of 0.83 and a standard error of calibration (SEC) of 0.70. There was no significant deviation from the actual BCS by NIR evaluation, while the difference was particularly distributed over a range of ±1.

In the second derivative spectra (Figure 4B), clear absorption peaks were observed at approximately 760 nm and 930 nm. The former peak was considered to contain information regarding red in the visible region, whereas the latter peak represented fat in the NIR region. There was also an absorption peak of myoglobin related to meat color at 700–1,050 nm [19], and the NIR evaluation may predict meat color from this information.

The visual evaluation of meat color is done using a 7-point scale with the BCS. Meat color that is too light or dark is not preferable, whereas a color of 3–5 is considered the 5th grade with the best meat color based on the grading standard (Table 1).

Some carcasses exhibit a meat color close to the intermediate color of each number. For example, for carcasses that are close to the intermediate color between No. 4 and No. 5, the rating members grade it as either No. 4 or No. 5, because it is not possible to add No. 4.5 as a decimal point. Because the BCS number is an integer for a natural phenomenon, it is possible that the BCS evaluation by the grading members can yield a measurement error of less than ±1. Therefore, it is important to predict BCS based on the number of carcasses that are within ±1 of the difference between the actual and predicted values [10]. Therefore, to verify the accuracy of the calibration model using unknown samples, we determined whether the difference between the actual and predicted BCS was within ±1.

### Accuracy verification of the Beef Color Standard calibration model

**Figure 6:**
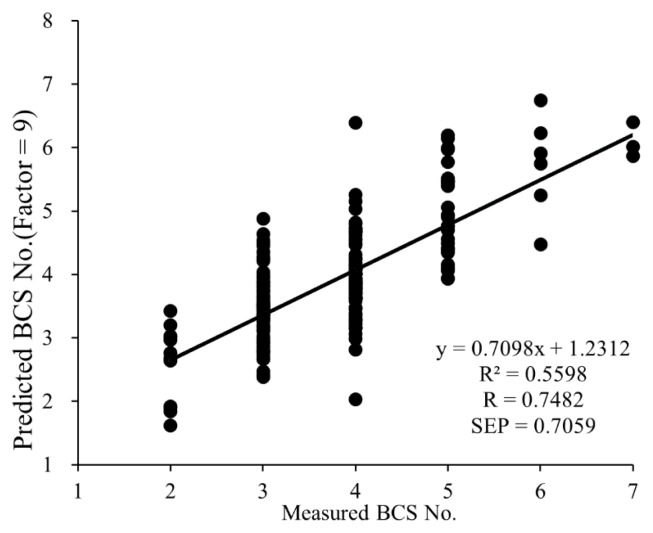
Measured and predicted Beef Color Standard (BCS) values in beef carcasses using the calibration model integrated into the handheld fiber optic near-infrared spectrometer. R^2^, coefficient of determination; R, correlation coefficient; SEP, standard error of prediction.

The accuracy of the calibration model (n = 827) by NIR evaluation was verified using 250 samples that were separate from those used for calibration model preparation (Figure 6). The correlation coefficient between the measured BCS value by visual evaluation and the predicted BCS value by the calibration model was 0.74, which is considered a relatively high value. The predicted standard error (SEP) of the predicted BCS value compared with the actual BCS value was 0.71, which was similar to the standard error of the calibration model. The RPD value of the calibration model is 2.4 or higher [16], which is considered a good indicator of its practicality for chemical analysis; however, the RPD value in the present study was 1.2. BCS judgment is an integer value, and a number below the decimal point is not required. Therefore, as mentioned above, the accuracy verification for this calibration model was performed using the probability that the difference between the actual and predicted BCS was ±1 (Table 2).

Of the 250 unknown samples, 101 were from JB, and none were from BCS No. 6 or 7. The probability that the difference between the measured the predicted BCS was within ±1 was 95.0%, whereas one carcass did not fall within ±1 for BCS Nos. 3 and 4. There were 102 samples for F1, and there were no samples with BCS No. 6 or 7, as observed in the JB carcasses. The difference between the measured and predicted BCS was within ±1 for all carcasses. However, in dairy cows, BCS 2–7 samples existed, and 43 of 47 samples were within ±1, with a probability of 93.6%. Including all varieties, the probability of a value within ±1 was 97.6%. Although there were no BCS No. 1 carcasses, only three No. 7 carcasses were used in this study. During BMS estimation by image analysis, the probability of an error between the actually measured and predicted BMS for JB Wagyu beef was ±1, which is 94.1% [9], and was 95.9% in another report [10]. In addition, although simple comparisons are impossible due to different evaluation methods employed, Kwon et al. [8] reported a prediction accuracy of 86% for Korean beef. Compared to the prediction accuracies of these marblings, the calibration model employed in this study could predict a wide range of BCSs with a relatively high degree of accuracy.

**Figure 7:**
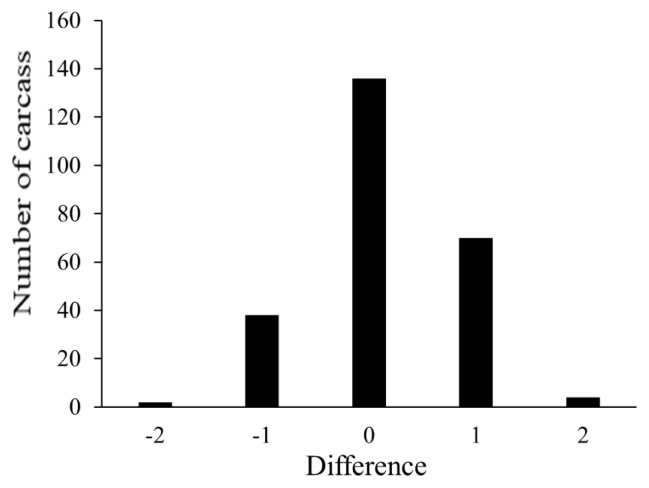
Difference between measured and predicted Beef Color Standard (BCS). Difference = Measured BCS − Predicted BCS.

The distribution of the difference between the measured and predicted BCS is shown in Figure 7. With zero difference, 136 of 250 samples had a predicted value—rounded to the nearest integer—that matched the actual measured value with a probability of 54.4%. There were 70 samples with the predicted value exceeding the actual value by 1 point, and 38 samples had a 1-point lower value. According to the scatter plot in Figure 6, there were many carcasses predicted to be 1 point higher than the measured BCS No. 3. Conversely, for BCS No. 3, very few carcasses predicted to be 1 point lower. In addition, there were a few carcasses predicted to be 1 point higher than BCS No. 5. The highest grade 5 was BCS number 3–5 (Table 1); thus, there were a few carcasses with lower grades according to the BCS prediction value by the calibration model.

Various chemical composition predictions using NIR have been used thus far, and NIR methods can be divided into two types [20]: those utilized for evaluating pretreatment, such as minced meat in a laboratory setting, and those for evaluating intact meat. Because the prediction of intact meat involves a more heterogeneous tissue, the prediction of sample uniformity by mincing is more accurate [20,21]. To predict in meat markets, the method must be non-destructive, whereas predictions by mincing are only for laboratory analysis [22]. In addition, high-spec large benchtop devices are often used in reports for laboratory analysis; however, they should be portable for practical use in meat markets. Estimation of chemical composition by portable NIR devices has not yet been reported in the livestock field [13,14,23,24].

Prieto et al. indicated the need to advance NIR prediction not only for chemical composition but also regarding physical properties [25]. NIR prediction of meat color, which is a physical property, may be divided into NIR prediction with or without a visible light range. For the former, the prediction accuracy of a* of the Lab parameters, which is highly related to meat color, is not often at a practical level [25,26]. Liu et al. [27] successfully predicted the Hunter parameters a, b, and E* for meat color using visible and NIR light. Hasan et al. [28] indicated that the accuracy of a* predictions is low only in the wavelength range of NIR without the visible light range, and variables in the wavelength range of 503–850 nm are important.

The wavelength range of the device used in the present study was 700–1,050 nm, which is considered as one of the reasons why visible and NIR light were able to accurately predict marbled beef. In addition, Prieto et al. [25] indicated that the high correlation with fat content results in predictability, although it is related to L*. Although the height of a* does not match the BCS during visual evaluation, this device, which has been put into practical use for measuring fat content, compensates for the effect of marbling, so it may improve the accuracy of the BCS measurement of marbled beef. If the device is improved to include a wavelength range of 500–700 nm, the prediction accuracy may be further improved.

The current visual evaluation by rating members is faster than the NIR prediction method. Because it is primarily conducted by multiple members, it is also the study objective, thereby ensuring that the NIR prediction method will not replace visual evaluation; however, the results of this study may be useful for researchers to evaluate meat color in the laboratory and to establish grading workshops.

In the future, there is a possibility that grading evaluations will be more efficient with less manpower. There are currently more opportunities to optically measure not only fat quality, but also the intramuscular fat content of the longissimus thoracis muscle. In such cases, the objective meat color evaluation value can be displayed at the same time by replacing the software. In addition, because the BCS judgment by the grader is done with the loin core, it may also be used to evaluate the color of the meat in other parts. Since more than 200 devices used in the present study have been sold, further applied studies and social implementation of various meat traits can be expected in the future.

## CONCLUSION

The correlation coefficient, coefficient of determination, and SEC were 0.83, 0.69, and 0.70, respectively, in the calibration model for estimating the BCS number by NIR. The probability that the difference between the predicted BCS and the actual BCS using the calibration model was within ±1 and ±0 was 97.6% and 54.4%, respectively. Therefore, the prediction of BCS numbers via the NIR method with high accuracy is possible.

## Figures and Tables

**Table 1:** Meat color classification among meat quality grades by the Japan Meat Grading Association

Grade		BCS No.	Polish
5	Very good	No. 3 to 5	Very good
4	Somewhat good	No. 2 to 6	Somewhat good
3	Standard	No. 1 to 6	Standard
2	Slightly inferior	No. 1 to 7	Slightly inferior
1	Inferior	Anything other than grade 5–2	

BCS, Beef Color Standard.

**Table 2:** Percentage of the Beef Color Standard (BCS) predicted value by NIR and the rating value within ±1 (per breed and BCS number)

BCS No.	JB			F1			Dairy cow			Total		
			
	n	Difference within ±1		n	Difference within ±1		n	Difference within ±1		n	Difference within ±1	

		n	Percentage		n	Percentage		n	Percentage		n	Percentage
No. 2	9	9	100.0	1	1	100.0	2	2	100	12	12	100.0
No. 3	50	49	92.0	47	47	100.0	2	0	0	99	96	97.0
No. 4	36	35	95.0	50	50	100.0	12	11	91.7	98	96	98.0
No. 5	6	6	100.0	4	4	100.0	22	22	100	32	32	100.0
No. 6							6	5	100	6	5	83.3
No. 7							3	3	100	3	3	100.0
	101	99	95.0	102	102	100	47	43	93.6	250	244	97.6

Difference = Measured BCS − Predicted BCS.

NIR, near infrared; JB, Japanese Black; F1, cross breed.

## Data Availability

Upon reasonable request, the datasets of this study can be available from the corresponding author.
